# Analyzing the Open/Deep Web to Better Understand the New/Novel Psychoactive Substances (NPS) Scenarios: Suggestions from CASSANDRA and NPS.Finder Research Projects

**DOI:** 10.3390/brainsci10030146

**Published:** 2020-03-04

**Authors:** Fabrizio Schifano

**Affiliations:** Psychopharmacology, Drug Misuse & Novel Psychoactive Substances Research Unit, University of Hertfordshire, Health Research Building, College Lane Campus, Hatfield AL10 9AB, UK; f.schifano@herts.ac.uk

**Keywords:** NPS, new psychoactive substances, drug misuse, deep web, open web, NPS.Finder

## 1. What are the New/Novel Psychoactive Substances (NPS): Definitions

New/novel psychoactive substances (NPS) are defined as new narcotic/psychotropic drugs which are not controlled by the United Nations’ 1961 Narcotic Drugs/1971 Psychotropic Substances conventions, but which may pose a public health threat [1]. However, ‘new’ can include a failed pharmaceutical or an old patent which has been ‘rediscovered’ and marketed for its potential use as a ‘recreational’ substance. Conversely, the term ‘novel’ can also express something newly created, or a compound that has come back into fashion after a period of absence from the recreational drug scene, or indeed a known NPS molecule being used in an innovative or unusual way, hence presenting with a ‘novelty’ appeal [1]. 

NPS are a reason of current/potential public health concern and typically include: synthetic cannabinoids, cathinone derivatives, psychedelic phenethylamines, novel stimulants, synthetic opioids, tryptamine derivatives, phencyclidine-like dissociatives, piperazines, GABA-A/B receptor agonists, a range of prescribed medications, psychoactive plants/herbs, and a large series of image- and performance-enhancing drugs (for a thorough review, see [2]). Users are typically attracted by these substances due to their intense psychoactive effects and likely lack of detection in routine drug screenings [3,4]. 

The accelerating speed of NPS appearance [1] is being associated with a range of key challenges, including: identifying how NPS are being created or ‘discovered’, revealing what their likely properties are and determining the adverse health effects, morbidity, or mortality that they cause. Because of this rapid NPS turnover, there might be now a ‘second generation of new substances’ brought about by changes to the NPS previously identified, with the aim of circumventing legal and regulatory responses and amend-ments as they appear [5].

## 2. What Are the Emerging NPS? How Many NPS Are Available? The Current European and International Approaches 

Both the European Monitoring Centre for Drugs and Drug Addiction (EMCDDA) and the United Nations Office on Drugs and Crime (UNODC) include an index NPS in their database only when the emerging NPS is seized, chemically analyzed and reported to them. Between 2009 and 2017, a total of 803 NPS were reported to the UNODC by 111 countries/territories. Conversely, by the end of 2017, the number of EMCDDA NPS was over 670, of which 632 were notified after 2004; most molecules were synthetic cannabinoids, synthetic cathinones, phenethylamine derivatives and synthetic opioids (for a thorough overview, see [3]). However, one could argue that the NPS scenario is much larger and more complex than that formally identified by EU/international agencies. 

The earliest of the possible appearance of a new substance might be evidenced on the deep, followed by a migration to the open, web [1]. Eventually, the NPS would move into ‘head shops’ and/or the ‘street’ market, then reported by formal early warning systems, and new legislation would be implemented to counter the index NPS/substance. Hence, an approach aiming at describing what is being discussed online by the web-based NPS enthusiasts ‘e-psychonauts’ has been considered as potentially useful to identify in advance the NPS availability, market and diffusion. In fact, the online NPS scenario, with its related concerns, typically predicts the real life NPS scenario [4]. Methods for online monitoring of the web developed by previous projects of our research group, such as Psychonaut [6], ReDNet [7], EU-MADNESS (http://www.eumadness.eu/) and Enhancing Police EPS/NPS project (http://www.npsproject.eu/) are currently being adapted and refined for deep-web monitoring. 

## 3. Monitoring the NPS-Related Hidden/Dark Areas of the Web: the CASSANDRA Project

The hidden-web drug marketplaces and crypto markets are increasingly being used for the anonymous sale of drugs, including NPS. Recent legislation, such as the May 2016 UK Psychoactive Substances Act, has been created to tackle NPS diffusion [8]. As commented in [9], although the Act may have achieved a reduction in UK-based online stores and offline retail stores, the easily tracked open-web drug transactions could be displaced by alternative routes such as street-level drug dealing or crypto markets. Conversely, within the ‘dark’ areas of the web, any transaction can be carried out with both the location and real identity of the parties being totally irrelevant. Further characteristics of appeal for interested customers include [5]: (a) payments are made using virtual currency (e.g., bitcoins); (b) subjects are required to accept an anonymous protocol (e.g., the Onion Router—Tor, or similar approaches), thereby decreasing the likelihood of the hidden servers being traced and identified; (c) use of an online assessment system, whereby the various parties can give their feedback about a given transaction, product or delivery.

Wadsworth et al. [9] elegantly explored the availability of individual NPS and vendors on the crypto markets. They collected data from 22 such markets that were accessed through Tor. Data collection took place bimonthly as part of the 2015-6 CASSANDRA project; seven snapshots were collected over 12 months, identifying 808 unique vendors who were selling 256 unique NPS. Overall, both the numbers of individual NPS and vendors increased overtime, by some 94% and 72%, respectively. Only around 1 out of 4 of the total number of NPS, and 1 out of 25 vendors, appeared in every snapshot over the 12-month observation period, whereas 21% of NPS and 45% of vendors only appeared once throughout the data collection. The majority of the NPS that were available in every snapshot were cathinones and phenethylamines. However, there was frequent turnover throughout the study, with some NPS having been available for purchase for a few months and then disappearing from availability. Wadsworth et al. [9] suggested that this NPS appearance/disappearance intriguing pattern may have mirrored changes in popularity within the overall NPS market, with fluctuations depending on both legality and ease of access issues. Other explanations would be related to the index NPS withdrawal from the market because of limited stock availability and/or because of negative customer feedback. 

## 4. Systematic Monitoring of the Web to Better Understand Current and Future Drug Scenarios: The NPS.finder Systematic Web Crawling Approach

Analyzing the information/data made available by e-psychonauts requires the management of ‘high-volume, high-velocity, and high-variety’ range of modifications in the NPS market, hence of ‘big data’ [10]. 

The use of automatic web crawler software such as the University of Hertfordshire (UK)-coordinated NPS.finder has allowed both scanning and identification activities of a constant flow of drug-related data, providing a nearly real-time update [11,12]. Current NPS.finder research project aims include: (a) identifying and describing the large number of novel/new psychoactive substances (NPS) available as identified from a range of psychonauts’, NPS-related, online sources; and (b) identifying a methodology which may be able to anticipate, for each class of NPS, the modifications of their availability levels in the real life scenarios.

So far, an excess of 4200 different NPS have been identified, mostly being phenethylamines and synthetic cannabimimetics [3,4]. To improve accuracy and provide a thorough evaluation of NPS use, however, further research should focus on an integrative model in which web-based analyses will be combined with more advanced research approaches. From this perspective, current related in silico, in vitro, and in vivo studies will hopefully provide important findings. It will then be possible to collect more theoretical/putative information relating to possible health risks (acute and chronic) physical/mental, morbidity, and mortality on a specified drug without waiting for its appearance [1]. Furthermore, understanding the pharmacological characteristics/potency of those NPS molecules which are of appeal for potential consumers will hopefully help in both predicting NPS diffusion and reducing the existing gap in knowledge between the web-based consumers and clinicians, hence facilitating the planning of ad hoc prevention strategies.

So far, the NPS.Finder has crawled on the open web only. Future studies by our group will be expanding drug searches on less accessible areas of the web, such as the deep web and the most elusive darknet [13]. A qualitative/netnographic approach [11] will be needed as well, to better assess the possible psychonauts’ preference between NPS analogues and their motivation for use. Furthermore, next NPS.Finder-based research will need to focus as well on specific, popular web languages, e.g., Chinese, Japanese and Arabic. 

## 5. Conclusions

It is here concluded that the open-, deep- and dark-web areas’ monitoring activities may well possess the potential to identify a wide range of novel/previously undescribed NPS. Better levels of misusing drugs’ clinical pharmacological-related knowledge are needed so that properly tailored management/treatment strategies and guidelines can be drawn up and made available. Finally, strengthening multidisciplinary collaboration between clinicians and bioinformatics may prove useful in better assessing the NPS-associated public health risks.
